# Cell and neuron densities in the primary motor cortex of primates

**DOI:** 10.3389/fncir.2013.00030

**Published:** 2013-02-27

**Authors:** Nicole A. Young, Christine E. Collins, Jon H. Kaas

**Affiliations:** Department of Psychology, Vanderbilt UniversityNashville, TN, USA

**Keywords:** M1, flow fractionator, isotropic fractionator, movement

## Abstract

Cell and neuron densities vary across the cortical sheet in a predictable manner across different primate species (Collins et al., 2010b). Primary motor cortex, M1, is characterized by lower neuron densities relative to other cortical areas. M1 contains a motor representation map of contralateral body parts from tail to tongue in a mediolateral sequence. Different functional movement representations within M1 likely require specialized microcircuitry for control of different body parts, and these differences in circuitry may be reflected by variation in cell and neuron densities. Here we determined cell and neuron densities for multiple sub-regions of M1 in six primate species, using the semi-automated flow fractionator method. The results verify previous reports of lower overall neuron densities in M1 compared to other parts of cortex in the six primate species examined. The most lateral regions of M1 that correspond to face and hand movement representations, are more neuron dense relative to medial locations in M1, which suggests differences in cortical circuitry within movement zones.

## Introduction

The cerebral cortex is a heterogeneous structure that contains multiple sensory and motor information-processing systems (e.g., Van Essen et al., 2011). A hallmark of the mammalian cerebral cortex is the regular arrangement of sensory and motor areas across its surface. Primary sensory areas are topographically organized to represent sensory receptor arrays and primary motor cortex has a general somatotopic organization of motor movement representations. It is reasonable to presume that numbers of cells and neurons in functionally distinct cortical areas vary according to information-processing demands. However, the majority of studies on this issue have only reported the total number of cells and neurons for the cerebral cortex as a whole (e.g. Pakkenberg and Gundersen, 1997; Christensen et al., 2007; Herculano-Houzel et al., 2008).

To date, only one study has detailed the total numbers of cells and neurons across the entire cortical expanse after dissection of the cortex into small tissue pieces (Collins et al., 2010b). This study not only demonstrated a clear, non-uniform distribution of cells and neurons across the cortex of all four primate species examined, it also illustrated a pattern of distribution of cells and neurons that was consistent across all of the primate species that were studied. The features of the typical primate pattern of cell and neuron distribution and number are (1) the highest cell and neuron densities are found in primary visual cortex, V1; (2) extrastriate cortical areas have relatively high cell and neuron densities; (3) primary auditory and somatosensory areas have relatively high cell and neuron densities compared to surrounding areas; and (4) motor cortex, M1, appears to have low neuron densities compared to other areas of cortex. These results are consistent with the earlier findings of Beaulieu and Colonnier (1989) who determined neuron number in the cortex below 1 mm^2^ of cortical surface in four visual areas, somatosensory area 3b, and two motor areas (4 gamma, 6a alpha) of the cortex of cats, and found that motor areas have the smallest number of neurons per column, while sensory areas contain more neurons, with the greatest number found in the binocular region of visual area 17. Skoglund et al. (1996) later investigated the number of neurons in primary motor, primary somatosensory and the second visual area in rats, and found significant differences in the number of neurons under a fixed amount of cortical surface area. They found that neuron density was highest in the second visual area (not V1), followed by primary somatosensory cortex. Primary motor cortex was the least neuron-dense cortical area. Despite the comprehensive evaluation of cortical cell and neuron number and distribution in the study by Collins and colleagues, the number of species examined in detail was limited and the dissection techniques used in that study had not been refined to allow evaluation of variation in cell and neuron density in different parts of topographically organized cortical areas, and in cases where functional areas were dissected from the cortex, areal boundaries were estimated from surface landmarks and sulcal patterns.

The present study was designed to examine the neuron densities in additional species of primates, to determine whether different representational zones within M1 have variable cell and neuron densities, and to compare estimated areal border locations with those determined electrophysiologically. We use the semi-automated flow fractionator method (Collins et al., 2010a; Young et al., 2012) to contrast M1 cell and neuron densities with the overall average densities across the cortex, and also to another specific primary cortical area, V1, to determine if M1 is contains relatively fewer neurons, as previously reported using other counting techniques. Here we also report estimates of neuron and total cell densities for different movement representation zones in M1 for three primate species. Variation in neuron and total cell densities within a sub-region of M1 may correspond to the type of movement produced in the sub-region.

## Materials and methods

### Tissue

Prosimian galago (*Otolemur garnetti*, *n* = 3), New World owl monkey (*Aotus nancymae*, *n* = 1), and squirrel monkey (*Saimiri sciuresis*, *n* = 1) brains were obtained from ongoing experiments of other investigators at Vanderbilt University. Galago and New World monkey brains were perfused with 0.1 MPBS. Old World macaque (*Macaca nemenstrina*, *n* = 2) and baboon (*Papio cynocephalus anubis*, *n* = 1) brains were purchased from the Washington National Primate Research Center. An additional baboon (*Papio hamadryas anubis*, *n* = 1) brain and a Hominid chimpanzee (*Pan troglodytes*, *n* = 1) brain were purchased from the Texas Biomedical Research Institute. These brains were perfused with 0.1 MPBS, and shipped to us overnight in the same solution. All brains were bisected and one cortical hemisphere was separated from the subcortical structures, the pia was removed and the sulci were opened to flatten the cortical sheet. The flat hemispheres were fixed within 4% paraformaldehyde (PFA). Macaque 2, however, remained intact and was post-fixed in 4% PFA. M1 was dissected from the flattened hemispheres and separated from the remaining cortex after viewing the flattened hemispheres on a light box to identify myelin-dense sensory areas that appear darker relative to surrounding cortical areas, which is an effective means to quickly visualize cortical areas for this method of dissection (see Collins et al., 2010b; Campi et al., 2011). Boundaries of M1 were identified relative to the estimated boundaries of these areas, sulcal landmarks, and in reference to previously published studies that identify boundaries of M1 and its internal organization (Gould et al., 1986; Huang et al., 1988; Waters et al., 1990; Huntley and Jones, 1991; Donoghue et al., 1992; Gaspar et al., 1992; Nudo et al., 1992; Stepniewska et al., 1993, 2005; Preuss et al., 1997; Jain et al., 2000; Qi et al., 2000, 2010; Wu et al., 2000; Fang et al., 2008; Wong and Kaas, 2010; Gharbawie et al., 2011; Kaas, 2012). M1 boundaries in the intact brain of baboon case 09–04 were identified according to intact sulcal landmarks and data from a previous report (Waters et al., 1990). In the galago, owl monkey, squirrel monkey, and baboon cases, the boundary of primary visual cortex (V1) is visible on the fixed brain surface, whether the cortex is flattened or not. Dissection cuts were placed along the visible, readily identifiable boundary. Remaining cortex in all species was dissected into approximately 5 × 5 mm cortical pieces. All cortical pieces were weighed, and surface areas were measured when possible using freely available NIH Image J software (NIH, Bethesda, MD, USA).

### Motor and somatosensory mapping of a galago

The primary motor map was derived for one galago (galago 3) using short-train intracortical microstimulation (ICMS). This mapping was undertaken to verify our estimation of M1 boundaries and its internal organization in comparison to estimates in other cases. Some sensory mapping was completed to further confirm the location of the caudal boundary of M1. Detailed methods for surgical preparation, and motor and sensory mapping, are described in previously published reports (see Wu et al., 2000; Wu and Kaas, 2003; Stepniewska et al., 2009; Qi et al., 2011). All surgical procedures were conducted in accordance with the National Institutes of Health Guide for the Care and Use of Laboratory Animals and with the approval and guidance of the Vanderbilt University Animal Care and Use Committee.

In brief, surgical mapping procedures were performed under isoflurane anesthesia. After the skull was opened, the dura was retracted and the cortex digitally photographed. Isoflurane anesthesia was replaced with ketamine hydrochloride diluted with physiological saline (1:4) delivered intravenously with an infusion pump to maintain a stable level of anesthesia (30–50 mg/kg/h). Ketamine did not profoundly suppress cortical responsiveness. The blood vessel pattern on the cortical surface was used to guide electrode penetrations, which were marked on a printed copy of the digital photograph. The frontal cortex was explored with ICMS to identify the locations of the representations of body movements in area M1. In the ICMS procedure a stimulating electrode is lowered into the cortex to cortical layer 5 and the minimal electrical current necessary to elicit body movements is delivered to identify the motor representations for the cortical motor area. The microstimulation currents were delivered in 60-ms trains, with a pulse duration of 0.2 ms, and a pulse frequency of 300 Hz, which are the optimal parameters for eliciting movement in primates and other mammals (see Gould et al., 1986; Preuss et al., 1992; Wu et al., 2000; Young et al., 2011), without tissue damage. Stimulation was delivered with a low-impedance tungsten microelectrode inserted perpendicular to the cortical surface to a depth of 1.5–1.8 mm. This depth was found to be optimal for eliciting responses in prosimian galagos (Wu et al., 2000). Stimulating currents were generated with a Master 8 stimulator (AMPI) with a biphasic stimulus isolator (Bak Electronics Inc.). The entire body of the animal was monitored for ICMS-evoked movements by two observers during the mapping session. The face representation area of M1 was defined by eliciting any movements involving the mouth, tongue, jaw, nose, ears, and eyelids. The forelimb representation area of M1 was defined by eliciting movements involving the shoulder, arm, elbow, wrist, and digits. The trunk representation included movements of the upper and middle torso. Movements were classified as hindlimb when movements of the tail, legs, foot, and toes were made. Somatosensory mapping was undertaken to better identify the caudal boundary of M1. Microelectrode penetrations were made in somatosensory cortex and the magnitudes of neuronal responses to tapping and manipulation of the body surface (face, forelimb, trunk, and hindlimb regions), as indicated by the acoustic strength of neuron firing audio output, were constantly evaluated as the microelectrode passed through the superficial to the middle layers of cortex (up to 1000 μm depth). For each penetration, the receptive field location, size, and stimulus preference to light touch, tapping, and joint movement stimuli at the site where the strongest evoked response occurred were recorded on the photograph of the cortical surface. Boundaries of M1 and S1, and identifiable movement representations areas within M1, were marked with fluororuby tracer (FR) immediately before sacrifice to estimate their location on the cortical surface. Following euthanasia, the brain was removed and the right cortical hemisphere was manually flattened. The FR landmarks were used to conservatively dissect ICMS-derived M1 representations from remaining cortex for processing with the flow fractionator (Young et al., 2012).

### Cell and neuron density estimates

The isotropic fractionator and flow fractionator cell and neuron counting methods were used to obtain cell and neuron estimates in cortical samples. Detailed processing steps for the isotropic fractionator and flow fractionator are described in previous reports (Herculano-Houzel and Lent, 2005; Collins et al., 2010a; Young et al., 2012). Cell and neuron estimates obtained by the isotropic fractionator and the flow fractionator are in excellent concordance (Collins et al., 2010a; Young et al., 2012), and therefore the two methods can be used interchangeably.

In brief, each cortical piece was homogenized using a glass Tenbroeck tissue grinder (Fisher Scientific) and a dissociation solution of sodium citrate and triton X-100 in distilled water. The resulting homogenized suspensions contained free-floating nuclei. The total suspension volumes were determined based on the sample density, resulting in suspension volumes between 2 and 6 ml. The total number of cells in a nuclear suspension was estimated using DNA staining with 4′,6-diamidino-2-phenylindole (DAPI) that fluoresces bright blue with ultraviolet excitation (460 nm emission). DAPI binds strongly to DNA and labels all nuclei in the suspension, regardless of cell type, thereby providing a means for determining cell number in the homogenized samples. The total cell number estimate includes all DAPI-positive cell types contained within the sample, including glia and endothelial cells, as well as neurons. Free-floating, DAPI-stained nuclei in samples from the main suspension were counted to estimate total cells using either fluorescence microscopy and a glass Neubauer counting chamber and matched coverslip (isotropic fractionator), or a fixed volume of 50 μl of Countbright absolute counting beads (Invitrogen) was added to the sample prior to evaluation using a Becton Dickson (BD) 5-laser LSR II flow cytometer equipped with a 355 nm laser and using BD™ FACSDiva v. 6.1.3 software (flow fractionator). The total number of neurons in a nuclear suspension was estimated by immunolabeling a sample of the main sample suspension for neuronal nuclei with the anti-NeuN antibody (anti-neuronal nuclear antigen) (Millipore, Inc.) to determine the percentage of the total nuclei (DAPI+) that are also NeuN-immunoreactive (NeuN-IR). All samples went through epitope retrieval, which consisted of 30 min in 0.2 M boric acid solution in an oven set at 70°C. After epitope retrieval, samples were washed once with PBS then re-suspended in PBS with primary antibody against NeuN added. Alexa Fluor 594 (AF594) goat anti-mouse IgG secondary antibody (Invitrogen, Inc.) was used to fluorescently tag NeuN-IR nuclei for counting on the fluorescence microscope (isotropic fractionator), and Alexa Fluor 647 (AF647) goat anti-mouse IgG secondary antibody (Invitrogen, Inc.) was used to estimate the proportion of NeuN-IR nuclei to the total population of DAPI+ nuclei on the flow cytometer. Detailed procedures for gating the flow cytometry data have been discussed elsewhere (see Collins et al., 2010a; Young et al., 2012). All flow cytometry experiments were conducted in the Vanderbilt University Medical Center Flow Cytometry Shared Resource.

## Results

**Figure 1:**
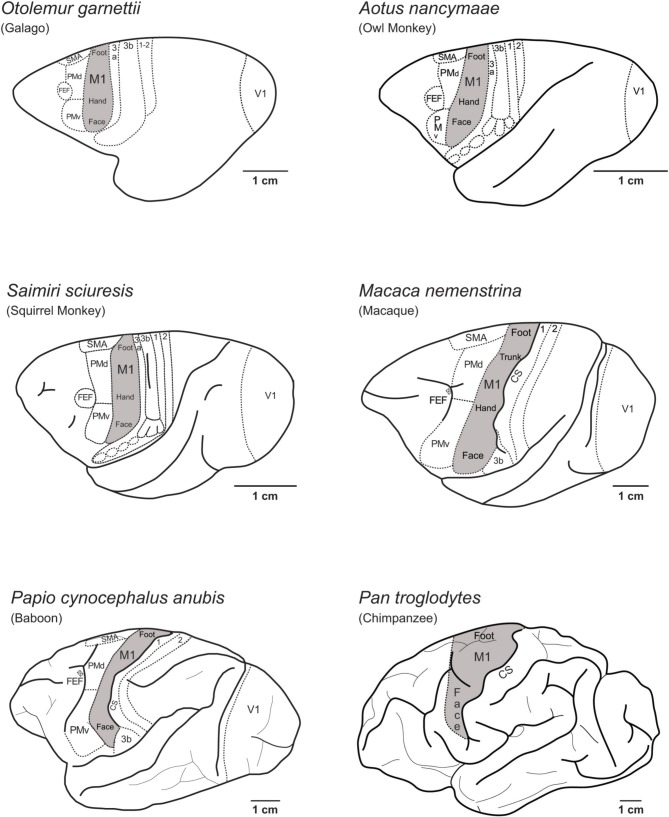
**The location of the primary motor cortex (M1) on the lateral view of the left cerebral hemisphere in several of the primate species discussed in this paper.** The location and internal organization of M1 in New world and Old World monkeys (Gould et al., 1986; Huang et al., 1988; Waters et al., 1990; Huntley and Jones, 1991; Donoghue et al., 1992; Nudo et al., 1992; Kaas, 2012) and prosimian galagos (Wu et al., 2000) consists of a fractured mosaic of smaller areas devoted to particular movements that are dispersed within a larger somatotopic framework of functional movement domains (see Kaas, 2012; Kaas et al., 2012 for review). Figure 1 shows the locations of M1 in prosimian galagos (Wu et al., 2000; Wong and Kaas, 2010), New World owl monkeys (Gould et al., 1986) and squirrel monkeys (Donoghue et al., 1992; Nudo et al., 1992), Old World macaques (Huntley and Jones, 1991; Preuss et al., 1997) and baboons (Waters et al., 1990), and Great Ape chimpanzees (Bailey et al., 1950). Hindlimb movement representations (foot) are located at the most dorsal-medial aspect of M1, and transitions to movements of the trunk of the body (trunk), forelimb and hand (hand), and finally face at the most ventral-lateral aspect of M1. Cortical areas located rostral to M1 include the dorsal (PMd) and ventral (PMv) premotor areas, the supplementary motor area (SMA), and frontal eye fields (FEF). Somatosensory areas of the anterior parietal cortex (3a, 3b, 1, 2) are caudal to M1. The location of the central sulcus (CS) is indicated in macaque, baboon, and chimpanzee.

Figure 1 illustrates the locations of M1 on the lateral aspect of the left cerebral hemisphere of the primate species examined in the present study, including prosimian galagos, New World owl monkeys and squirrel monkeys, Old World macaque monkeys and baboons and a Hominid chimpanzee. The location and organization of M1, and the rest of the cortical motor areas, are similar across these species despite differences in the size of the cortex.

### Prosimian galagos

**Figure 2:**
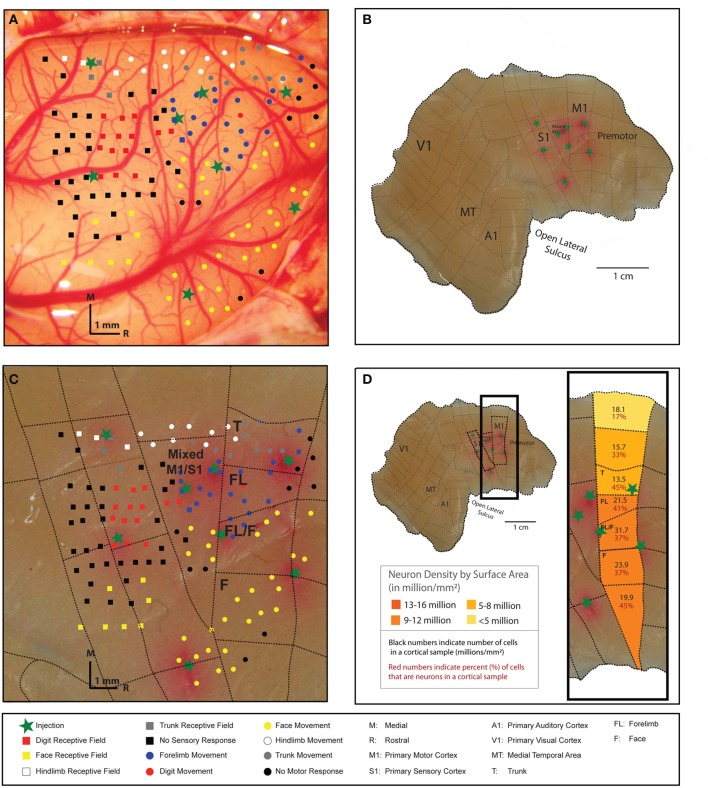
**The right cortical hemisphere of a galago (galago 3) was exposed, and movements were evoked at cortical sites in M1 with 60-ms microstimulation trains delivered to layer 5 (A).** The locations of stimulation sites where movements were elicited are indicated by color-coded dots on the cortical surface. Somatosensory mapping of receptive fields (layer 4) responsive to tactile stimulation are indicated by color-coded squares on the cortical surface. Somatosensory mapping was undertaken to better identify the caudal boundary of M1. Boundaries of M1 and S1, and identifiable movement representations areas within M1, were marked with a fluororuby tracer (FR) immediately before sacrifice to estimate their location on the cortical surface (green stars). Following sacrifice, the brain was removed and the right cortical hemisphere was manually flattened **(B)**. The FR-indicated areas were used as guidelines to conservatively dissect microstimulation-derived M1representations from remaining cortex. Dashed lines indicate dissections lines that resulted in 84 pieces for the entire cortical hemisphere. The dissection resulted in four pieces identified as M1 by microstimulation mapping **(C)**. Areas dedicated to movements of the trunk, forelimb, and face were readily identified and efforts were made to dissect along these movement representations boundaries within M1. Each M1 piece, as well as cortical pieces medial and lateral to M1, had cell and neuron densities estimated using the flow fractionator method **(D)**. The most lateral aspect of M1, which includes facial movement representations, is the most neuron dense area.

In the three galagos analyzed here, the total surface area of the flat cortex was 1782 mm^2^ (galago 1), 1850 mm^2^ (galago 2), and 1817 mm^2^ (galago 3). The total cortex weight for these individuals is also very consistent at 2.86, 2.87, and 2.37 g for galagos 1, 2, and 3, respectively. M1 was identified and dissected in galagos 1 and 2 by visual identification on a lightbox using myelination patterns as reference. Galago 3 (case 10–41) had motor map dissections borders determined by ICMS mapping of M1 (Figure 2). Our methods for M1 identification and dissection (lightbox vs. mapping) was shown to produce consistent M1 cell and neuron estimates across individuals, and therefore we believe both dissection methods can be used to accurately identify cortical areas. For example, while the total surface areas for galago 3 (55 mm^2^) and galago 2 (78 mm^2^) slightly varied, the mapped galago 3 had a total number of 12.7 million cells and 4.9 million neurons within the defined M1 area, while the lightbox-dissected galago 2 had a total number of 11.0 million cells and 4.5 million neurons within M1.

Table 1 shows the results averaged for all three galagos. In galagos, M1 comprised approximately 3.1% of the total cortical mass, and almost 3% of the total cortical surface area. The average cell density in M1 was 118 million cells/g or 181,000 cells per mm^2^ of cortical surface area. We found that the average proportion of DAPI-labeled nuclei that were also NeuN-IR was 32.2%, meaning that 32.2% of nuclei in M1 were identified as neuronal. This resulted in the average neuron density in M1 of 29 million neurons/g or 68,742 neurons under 1 mm^2^ of cortical surface. To determine the magnitude of the cell and neuron density difference between M1 and V1 within each case, we calculated the average difference within a species for comparison with other species. The overall cortical cell density, averaged across all cortical areas and regions, including M1 and V1, in all three galagos, was 149 million cells/g or 205,398 cells per mm^2^ of cortical surface. In contrast with another primary cortical area, the average cell density in V1 was 241 million cells/g or 297,479 cells per mm^2^, the highest cell density in the cortex. The overall cortical neuron density across all areas was 64 million neurons/g or 86,231 neurons per mm^2^ of surface area. The average neuron density in V1 in galagos was 146 million neurons/g or 182,208 neurons per mm^2^ of surface area, approximately five times higher than the neuron density in M1 (29 million neurons/g and 68,742 neurons/mm^2^ of cortical surface).

**Table 1:** **Summary of neuron and cell density in primary motor cortex by mass and surface area**.

**Species**	**Mass (g)**	**Percent total mass**	**Area (mm^2^)**	**Percent total area**	**Cell density (millions)**		**Percent neurons in M1 (%)**	**Neuron density (millions)**		**Percent neuron difference from total average (%)**
					**Cells/g**	**Cells/mm^2^**		**Neurons/g**	**Neurons/mm^2^**	
*Otolemur garnettii* (*n* = 3)	0.065 ± 0.02	3.13 ± 0.03	43.22 ± 12.70	2.78 ± 0.82	118.84 ± 17.72	0.181 ± 0.038	32.2 ± 5.9	29.04 ± 12.32	0.068 ± 0.041	−7.1 ± 0.2
*Aotus nancymaae* (*n* = 1)	0.267	5.12	221.83	8.6	114.91	0.138	36.0	41.37	0.050	−3.1
*Saimiri sciuresis* (*n* = 1)	0.344	3.11	125.1	2.57	91.39	0.251	33.7	30.00	0.082	−21.3
*Macaca nemenstrina* (*n* = 2)	1.381 ± 0.160	N/A	N/A	2.20	75.06 ± 10.14	N/A	30.9 ± 5.7	23.51 ± 0.49	N/A	N/A
*Papio cynocephalus anubis* (*n* = 1)	2.102	4.20	653.8	3.52	80.78	0.260	34.8	28.11	0.090	−14.93
*Papio hamadryas anubis* (*n* = 1)	2.000	3.55	636.4	2.72	72.62	0.228	38.6	28.10	0.088	−12.4
*Pan troglodytes* (*n* = 1)	7.520	N/A	2700	N/A	73.01	0.203	27.0	19.30	0.055	N/A

*All values are reported as mean ± standard deviation where applicable. The n value refers the number of cortical hemispheres available for study. Percent Total Mass refers to the percentage of cortical mass dedicated to M1 within the mass of the cortical hemisphere. Percent Total Area refers to the percentage of cortical area dedicated to M1 across the entire cortical hemisphere. Percent Neuron Difference for Total Average refers to the Percent Neurons in M1 minus the overall percent of neurons averaged across the entire cortical hemisphere*.

The M1 map derived by ICMS in galago 3 clearly illustrated a mediolateral pattern of the movement map from hindlimb to trunk, forelimb, and face and the boundaries between those movement representations (Figure 2A). Each representational zone in M1 was dissected in an effort to evaluate the mediolateral organization of the movement map. The dashed lines in Figures 2B,C show where the dissection cuts were placed relative to the physiological motor map. Figure 2D illustrates the cell and neuron estimates for each sample according to its location and corresponding movement representation in M1. The data illustrated in the figure show that the more lateral regions of M1, which correspond to the face area, contain a higher density of neurons per mm^2^ of surface area than medial M1.

### Old world primates

One macaque cortical hemisphere used in this study was manually flattened (macaque 1), while the cortical hemisphere of the other macaque brain (macaque 2) remained intact, thus surface area measurements were unavailable for macaque 2. The total surface area of the flattened cortical hemisphere was 15,200 mm^2^ (macaque 1) and weighed 116.1 g. M1 was visualized and dissected using the lightbox method for the flat cortical hemisphere in addition to the proximity to sulcal landmarks, while M1 in the intact cortical hemisphere (macaque 2) was estimated using sulcal patterns as landmarks based on previous reports (McGuinness et al., 1980; Wise and Tanji, 1981; Sessle and Wiesendanger, 1982; Huntley and Jones, 1991; Qi et al., 2000, 2010). Table 1 shows that the average M1 cell density in macaques is 75 million cells/g. The M1 cell density for macaque 1 alone was 84.1 million cells/g and the total cell density averaged across the entire cortex in case macaque 1 was 100 million cells/g. We found that the average proportion of DAPI-labeled nuclei that were also NeuN-IR was 30.9% in macaque monkeys. The average neuron density in M1 was 23.5 million neurons/g for macaques. The M1 neuron density for macaque 1 alone was 22.3 million neurons/g. When averaged across all areas of cortex in macaque 1, the overall neuron density was 48.0 million neurons/g.

For the baboons analyzed in this study, the total surface area of the flat cortex was 18,577 mm^2^ (baboon 1), and 23,400 mm^2^ (baboon 2). The total cortex weight for these individuals was fairly consistent at 50.0 g in baboon 1 and 56.4 g in baboon 2. M1 was identified and dissected in both baboon cases by visual identification on a lightbox using myelination patterns using sulcal landmarks as reference. Table 1 shows the summary of M1 data in both individuals. M1 comprised 3.55% (baboon 2) and 4.20% (baboon 1) of the total cortical mass, and 2.72% (baboon 2) and 3.52% (baboon 1) of the total cortical area. The average cell density in M1 was 80 million cells/g or 26,000 cells per mm^2^ of cortical surface for baboon 1, and 72.6 million cells/g or 22,800 cells per mm^2^ for baboon 2. The average proportion of DAPI-labeled nuclei that we found to also be NeuN-IR was 34.8% in baboon 1 and 38.6% in baboon 2, therefore the M1 neuron densities in these cases were 28.11 million neurons/g (90,000 neurons/mm^2^) and 28.10 million/g (88,000 neurons/mm^2^), respectively. These results were highly consistent across the two individuals.

We compared the M1 density averages to the overall cortical density average, and also compared them to the density averages for a readily identifiable primary sensory cortical area, V1 (primary visual cortex). The overall cortical cell density, averaged across all cortical areas and regions was 95.9 million cells/g or 251,603 cells per mm^2^ (baboon 1) and 78.3 million cells/g or 183,441 cells per mm^2^ (baboon 2). The neuron fraction across the cortex was 51% (baboon 1) and 54% (baboon 2), therefore the overall neuron density across the cortex was 51.1 million neurons/g or 128,950 neurons per mm^2^ and 42.9 million neurons/g or 97,005 million neurons per mm^2^, respectively. To contrast the M1 cell and neuron density data with a primary sensory cortical area, the average cell density in V1 was 144 million cells/g or 283,307 cells per mm^2^ (baboon 1) and 139 million cells/g or 223,456 cells per mm^2^ (baboon 2). In V1, the fraction of DAPI-labeled nuclei that were also NeuN-IR was 75% (baboon 1) and 72% (baboon 2), therefore the V1 neuron densities in each case were 109 million neurons/g or 210,479 neurons per mm^2^ and 101 million neurons/g or 163,100 neurons per mm^2^, respectively. Cell and neuron densities in M1 were lower than the overall cortical average, and they were also much lower than cell and neuron density estimates in V1. In this data set, we report the baboon data separately as each case was a hybrid species of baboon, however, there was remarkable consistency in the overall, M1 and V1 data between the two cases in cell and neuron densities, showing a roughly 3-fold decrease in the cell density in M1 compared to V1, and an approximately 5-fold difference in neuron density between M1 and V1.

**Figure 3:**
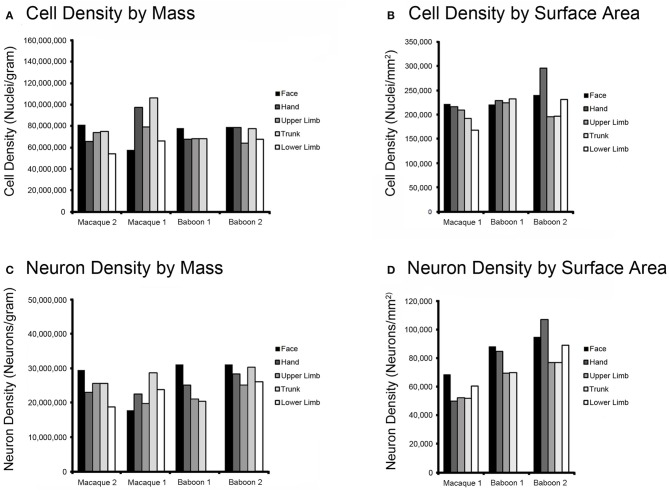
**Histograms of cell and neuron density in M1 of Old World monkeys.** Movement representation boundaries within M1 were estimated, the motor cortex was dissected along those boundaries, and cell and neuron densities were obtained using the flow fractionator. The results show that distribution of cells by mass **(A)** and by surface area **(B)** does not vary substantially within M1 in either the macaque (macaque 1; macaque 2) or baboon (baboon 1; baboon 2). Neuron density by mass **(C)** and surface area **(D)** shows a trend toward the most lateral aspect within M1, which includes face and hand movement representations, as being the most neuron dense area in both species. Please note that macaque 2 was an intact dissection and, as a result, cortical surface area could not be accurately estimated.

In each baboon and macaque case, we estimated the locations of M1 movement representations and dissected areas according to those estimates. Without electrophysiological data to support our boundary estimates, we assigned dissected M1 cortical pieces to the likely lower limb, trunk, upper limb, hand, and face representations in a mediolateral sequence. The data in Figure 3 shows the distribution of cells and neurons within these representations for each individual, in a lateral-to-medial sequence from left to right. The neuron densities was found be higher in lateral parts of M1, corresponding to the face and hand representations, than in medial M1 (Figure 3D).

### Owl monkey, squirrel monkey, and chimpanzee

The total surface area of the flattened owl monkey cortex was 2000 mm^2^ with a total weight of 5.21 g. The boundaries of M1 were estimated according to myelination patterns and cortical landmarks and was dissected and processed as a single sample. M1 comprised 5.12% of the total mass of the cortex and 8.6% of the total cortical surface area. The M1 cell and neuron density by weight are approximately 115 million cells/g and 41 million neurons/gram of cortical tissue. The M1 cell and neuron densities per mm^2^ of cortical surface are 138,000 cells/mm^2^ and 50,000 neurons/mm^2^. The overall cell density averaged across the entire cortex was 139 million cells/g or 276,595 cells per mm^2^. The overall proportion of neurons was 39%, compared to the M1 neuron proportion of 36%, resulting in an overall neuron density of 55.6 million neurons/g or 106,227 neurons per mm^2^. The V1 cell density in this case was 165 million cells/g or 270,703 cells per mm^2^. The proportion of cells in V1 that were NeuN-IR was 58%, resulting in a neuron density of 96.7 million neurons/g or 158,554 neurons per mm^2^ of primary visual cortex. V1 has approximately a two-fold higher neuron density than M1.

The flattened squirrel monkey cortex had a total surface area of 4900 mm^2^ and weighted 11.1 g. M1 comprised 3.11% of the total mass of the cortex and 2.57% of the total cortical surface area. Cell and neuron densities by weight are 91 million cells/g and 30 million neurons/g. Cell and neuron densities by surface area are 251,000 cells/mm^2^ and 82,000 neurons/mm^2^ of cortical surface area (Table 1). The overall cell density averaged across the entire cortex was 134 million cells/g or 279,862 cells per mm^2^. With an overall neuron fraction of 55%, the overall cortical neuron density was 79.5 million neurons/g or 159,457 neurons per mm^2^. V1 cell density was 202 million cells/g or 345,197 cells per mm^2^. With a V1 neuron fraction of 75%, this resulted in a neuron density of 152 million neurons/g or 255,897 neurons per mm^2^ of primary visual cortex. M1 neuron density is approximately five times lower than the neuron density in V1, and over two times lower than average cortical density.

In the chimpanzee, M1 weighted 7.25 g and had a surface area of 2700 mm^2^. The cell and neuron densities for M1 are 73 million cells/g and 19 million neurons/g. The cell and neuron densities for M1 by surface area are 203,000 cells/mm^2^ and 55,000 neurons/mm^2^ (Table 1). M1 contains about 27% neurons overall. V1 data are not yet available for this species.

## Discussion

Here we demonstrate that tissue boundaries for cortical areal dissections are readily determined by electrophysiological mapping, and that estimates of areal boundaries based on cortical landmarks also result in reasonably accurate dissections. The average percentage of NeuN-immunoreactive cells in M1 was fairly consistent across all six species of primates reported here, ranging from approximately 27% neurons in the chimpanzee to 36% neurons in the baboon. From the present dataset, it appears that primary motor cortex is approximately 2–5 times less neuron-dense than primary visual cortex and 1.4–2.7 times less dense than cortex overall in the species examined here. Four of the five species examined here showed a 5-fold difference in M1:V1 neuron density, with the exception being the nocturnal owl monkey that had a two-fold difference. The owl monkey, however, is the only species examined here to have an overall neuron density in V1 that is less than 100 million neurons/g of cortical tissue, making it unusual in comparison to the other primates. In species where it was possible to dissect M1 according to motor representations, there appears to be a trend toward a higher density of neurons in more lateral regions of M1, representing hand and face, compared to more medial areas representing trunk and hindlimb. These data, however, are based on one case where representational boundaries were defined electrophsyiologically and four other cases in which the boundaries were approximated using cortical landmarks.

### Cells and neuron densities in M1 in primates

The locations and organizations of M1 are similar across the six species of primates we have examined, and between individuals within a species, which suggests that the primate pattern of primary motor cortex emerged early in primate evolution, and has been conserved in the prosimian and simian branches within the primate order. We have shown that the proportion of mass and surface area dedicated to M1 remains relatively constant across species, despite the roughly 15-fold variation in total cortical surface area across the species examined.

## Architecture and neuron density

In a previous study, we demonstrated that primate M1 has a distinctively lower neuron density relative to other areas and regions of cortex and that the primate cortex is not uniform in its distribution of neurons across areas and regions (Collins et al., 2010b). These results are consistent with those of other reports in which the lowest neuron densities in the cortex reported for cats and rats were also in the primary motor area (Beaulieu and Colonnier, 1989; Skoglund et al., 1996). Our data extend these findings to primates. In addition, our findings illustrating variations in neuron density by representational zone in M1 are analogous to the previous finding of the highest neuron density in V1 of cats, occurring in the binocular representational zone (Beaulieu and Colonnier, 1989). In contrast to these consistent findings for M1 across rats, cats and a range of primates, a recent report including several species reported similar neuron densities for all cortical areas, including M1, except for V1 of primates (Carlo and Stevens, 2013). This study basically repeated the earlier observations of Rockel et al. (1980). We do not know the reasons for such contradictory findings, except to note that both of the studies used extremely sparse sampling, and the methods for identifying cortical areas were unclear.

One reason for a real differences in neuron density appears to be layer 4. The neuron dense layer of tightly packed, small neurons is remarkably thin and nearly indistinguishable in primary motor cortex, M1. Beaulieu and Colonnier (1989) concluded that areas in cats with the highest neuron densities tended to be sensory areas with a wide, neuron-dense layer IV, whereas motor areas had lower neuron densities and a significantly reduced layer 4. Variations in neuron densities across areas and regions of the cortex have been attributed to differences in developmental programs, with primary sensory areas having higher neuron densities (Dehay and Kennedy, 2007) and rostral portions of the cerebral cortex generally having a lower overall neuron density relative to more caudal cortical areas (Cahalane et al., 2012).

In addition to having a thin or missing layer of small granule cells, M1 is characterized by some of the largest of pyramidal cells (e.g., Stepniewska et al., 1993; Preuss et al., 1996, 1997). The sizes of pyramidal cells also differ across cortical areas (e.g., see Elston and Rosa, 1997; Elston and Rockland, 2002; Elston et al., 2005a,b; Bianchi et al., 2012) in ways that likely account for different functional capabilities. For example, spine densities on the basal dendritic arbors of pyramidal neurons in layer III of granular prefrontal cortex (gPFC) are as much as 16 times greater than spine densities on layer III pyramidal cells in V1 (Elston et al., 2006). These large neurons are more densely interconnected and potentially sum more sources of inputs within their more complex dendritic arbors (Elston, 2007). In general, small cells are activated by few inputs and thereby preserve information, while large pyramidal neurons sum many inputs and have integrated functions (Kaas, 2000).

The agranular and dysgranular cortex of M1 is comprised mostly of large pyramidal neurons in prominent layers 3 and 5, with some pyramidal cells in layer 5 being Betz cells, the largest neurons in the cortex, with diameters as large as 100 microns. Given the relatively lower density of neurons in primary motor cortex in the primates examined here, particularly in relation to the extremely high neuron densities in V1 where the neurons are extremely small, it seems reasonable to surmise that neuron density and neuron size may co-vary. However, previous research has examined the relationship between soma size and dendritic arbor size, concluding that the largest cells do not necessarily have the largest dendritic arbors (Elston and Rockland, 2002). Moreover, the spine density of neurons in M1 is significantly higher than that in V1 layer III pyramidal cells, but is lower than in pyramidal cells in layer III of premotor area 6 (Elston and Rockland, 2002; Elston, 2007).

### Heterogeneity within M1 in primates

We have previously demonstrated that M1 in primates is a region that, as a whole, is neuron sparse relative to the rest of cortex (Collins et al., 2010b). Here we demonstrate that it is also a heterogeneous structure that varies in neuron density across different representational zones, with lower neuron densities in medial M1 and higher neuron densities in lateral M1.

More medial M1 representing movements of the lower body is also more myelinated, possibly because corticospinal projections of the lower body movement representations in medial M1 are more heavily myelinated because they traverse longer distances to reach their more distant spinal cord targets (Glasser and Van Essen, 2011). Heavier myelination, in combination with a larger axon, would speed conductance to long-range targets. Hand and face body movement representations of lateral M1 would be less heavily myelinated due to closer proximity of their targets. In general, neurons with longer, thicker axons have larger cell bodies that reduce overall neuron densities.

Another possibility is that the higher neuron densities in lateral M1 reflect higher numbers of modulatory GABAergic interneurons. During the execution of voluntary movements, fast-spiking parvalbumin (PV)-expressing GABAergic interneurons are active in motor cortex, which suggests that they play a role in shaping ongoing movements (Isomura et al., 2009). The distribution and pattern of GABAergic immunoreacitve neurons in the visual areas processing areas (DeFelipe et al., 1999), and prefrontal cortex (Elston and Gonzalez-Albo, 2003) has been thought to vary with regional specializations related to information processing demands. The M1 face representation in hominids has a greater number of PV-immunoreactive neurons relative to Old World species, and this modification in face representation microcircuitry may be involved in supporting sophisticated coordination of facial muscles (Sherwood et al., 2004). A higher number of modulatory interneurons in face and hand representations may reflect an increased capacity for fine motor control, allowing the social advantage of better and increased skill in manipulating the external environment with the hands. The distribution of GABAergic subtypes have been characterized within specific movement representations and compared across species (Sherwood et al., 2003, 2004) or across other cortical areas outside M1 (Sherwood et al., 2010), but no study has yet examined the GABAergic neuron distribution within the mediolateral sequence of M1 movement representations.

### Conflict of interest statement

The authors declare that the research was conducted in the absence of any commercial or financial relationships that could be construed as a potential conflict of interest.
